# On a bivariate spectral relaxation method for unsteady magneto-hydrodynamic flow in porous media

**DOI:** 10.1186/s40064-016-2053-4

**Published:** 2016-04-14

**Authors:** Vusi M. Magagula, Sandile S. Motsa, Precious Sibanda, Phumlani G. Dlamini

**Affiliations:** School of Mathematics, Statistics and Computer Science, University of KwaZulu-Natal, Private Bag X01, Scottsville, Pietermaritzburg, 3209 South Africa; Department of Applied Physics and Engineering Mathematics, University of Johannesburg, P.O. Box 17011, Doornfontein, Johannesburg, 2028 South Africa

**Keywords:** Bivariate Spectral relaxation method, Magneto-hydrodynamic flow, Porous media, Primary 65N35, 76W99

## Abstract

The paper presents a significant improvement to the implementation of the spectral relaxation method (SRM) for solving nonlinear partial differential equations that arise in the modelling of fluid flow problems. Previously the SRM utilized the spectral method to discretize derivatives in space and finite differences to discretize in time. In this work we seek to improve the performance of the SRM by applying the spectral method to discretize derivatives in both space and time variables. The new approach combines the relaxation scheme of the SRM, bivariate Lagrange interpolation as well as the Chebyshev spectral collocation method. The technique is tested on a system of four nonlinear partial differential equations that model unsteady three-dimensional magneto-hydrodynamic flow and mass transfer in a porous medium. Computed solutions are compared with previously published results obtained using the SRM, the spectral quasilinearization method and the Keller-box method. There is clear evidence that the new approach produces results that as good as, if not better than published results determined using the other methods. The main advantage of the new approach is that it offers better accuracy on coarser grids which significantly improves the computational speed of the method. The technique also leads to faster convergence to the required solution.

## Introduction

This work describes a new approach to the solution of a system of four partial differential equations that model the flow of unsteady three-dimensional magneto-hydrodynamic flow and mass transfer in porous media. As reported in Hayat et al. (2010), such equations arise in many applications including the aerodynamic extrusion of plastic sheets, the cooling of metallic sheets in a cooling bath and the manufacture of artificial film and fibers. Due to these important applications, many researchers have dedicated time and effort in studying these kind of problems and finding their solutions. The particular model equations considered in this work have been solved in Hayat et al. (2010) using the homotopy analysis method (HAM) and more recently, by Motsa et al. (2014a) using the spectral relaxation method (SRM) and the spectral quasilinearization method (SQLM). The HAM has been used extensively by researchers working on such problems Abbas et al. (2008), Ahmad et al. (2008), Ali and Mehmood (2008), Mehmood et al. (2008), Alizadeh-Pahlavan and Sadeghy (2009), Fan et al. (2010), Xu et al. (2007), You et al. (2010). It is an analytic method for approximating solutions of differential equations developed by Liao (2012). The homotopy analysis method is an analytic method where accuracy and convergence are achieved by increasing the number of terms of the solution series. In some cases, such as when a large embedded physical parameter multiplies the nonlinear terms, far too many terms may be required to give accurate results. Retaining too many terms in the solution series is cumbersome, even with the use of symbolic computing software. The use of the HAM further depends on other arbitrarily introduced parameters such as the convergence controlling parameter which must be carefully selected through a separate procedure.

A popular numerical method used by many researchers to solve unsteady boundary layer flow problems is the Keller-box method Ali et al. (2010a, b), Lok et al. (2010), Nazar et al. (2004a, b). The Keller-box method is a finite difference based implicit numerical scheme which was developed by Cebeci and Bradshaw (1984). Recently, Motsa et al. (2014a, b) used spectral based relaxation and quasilinearization schemes to solve unsteady boundary layer problems. These schemes are accurate, easy to implement and are computationally efficient. As observed in Motsa et al. (2014a), the limitation of the spectral quasilinearization method is that the coupled high-order system of differential equations may often lead to very large systems of algebraic equations that may require significant computing resources. In addition, the actual process of developing the solution algorithm is time-consuming in comparison to SRM. This is because with SQLM, the process begins with the quasi-linearization step whereas with SRM the iteration scheme is obtained directly by requiring some terms to be evaluated at the current iteration and others at the previous iteration. The SRM works much like the familiar Gauss-Seidel iteration by decoupling a system of non-linear PDEs into a system of linear PDEs which are then solved in succession. Consequently, the SRM is easy to implement and computationally efficient.

Both the original SRM and SQLM used in Motsa et al. (2014a) use finite differences to discretize derivatives in time. This is a disadvantage because finite difference schemes are known to converge slower than spectral methods. The use of finite differences effectively nullifies the benefits of fast convergence when spectral collocation is used to discretize in space. Furthermore, finite differences require fine grids with very small step sizes to guarantee accuracy, hence there is a huge computation time overhead each time the grid is refined. This paper provides a different approach to the implementation of the spectral relaxation method introduced in Motsa et al. (2014a). The innovation is that the spectral collocation method is used to discretize derivatives in both space and time. As a result, there are uniform convergence benefits in both directions. The scheme uses fewer grid points in space and time and thus it converges very fast. We refer to the improved SRM as the bivariate interpolation spectral relaxation method (BI-SRM). To test the viability of this innovation as a solution method, we have solved the coupled system of third and second order partial differential equations that describe a boundary-layer system. A careful comparison of the new results is made with the earlier SRM, SQLM and the Keller-box results reported in Motsa et al. (2014a). We particularly compare the computational times for the different methods to reach the same level of accuracy.

## Model equations

We consider the unsteady and three-dimensional flow of a viscous fluid over a stretching surface investigated by Hayat et al. (2010). The fluid is electrically conducting in the presence of a constant applied magnetic field ${\text {B}}_0$. The induced magnetic field is neglected under the assumption of a small magnetic Reynolds number. The flow is governed by the following four dimensionless partial differential equations

1 $$\begin{aligned}&f''' + (1-\xi )\left( \frac{\eta }{2}f'' - \xi \frac{\partial {f'}}{\partial {\xi }}\right) + \xi \left[ (f+g)f'' - (f')^2 - M^2f' - \lambda f'\right] = 0, \end{aligned}$$

2 $$\begin{aligned}&g''' + (1-\xi )\left( \frac{\eta }{2}g'' - \xi \frac{\partial {g'}}{\partial {\xi }}\right) + \xi \left[ (f+g)g'' - (g')^2 - M^2g' - \lambda g'\right] = 0, \end{aligned}$$

3 $$\begin{aligned}&\theta '' + Pr(1-\xi )\left( \frac{\eta }{2}\theta ' - \xi \frac{\partial {\theta }}{\partial {\xi }}\right) + Pr\xi (f+g)\theta ' = 0, \nonumber \\&\phi '' + Sc(1-\xi )\left( \frac{\eta }{2}\phi ' - \xi \frac{\partial {\phi }}{\partial {\xi }}\right) + Sc\xi (f+g)\phi ' - \gamma Sc\xi \phi = 0 \end{aligned}$$

with the following boundary conditions

4 $$\begin{aligned}&f(\xi ,0) = g(\xi ,0) = 0, \;\;\; f'(\xi ,0) = \theta (\xi ,0) = \phi (\xi ,0) = 1, \end{aligned}$$

5 $$\begin{aligned}&f'(\xi ,\infty ) = g'(\xi ,\infty ) =\theta (\xi ,\infty ) =\phi (\xi ,\infty ) = 0, \end{aligned}$$

6 $$\begin{aligned}&g'(\xi ,0) = c \end{aligned}$$

In the above equations prime denotes the derivative with respect to $\eta$, *c* the stretching parameter is a positive constant. *M* is the local Hartman number, $\lambda$ the local porosity parameter, *Sc* the Schmidt number, *Pr* the Prandtl number and $\gamma$ the chemical reaction parameter. The initial unsteady solution can be found exactly by setting $\xi = 0$ in the above equations and solving the resulting equations. The closed form analytical solutions are given by

7 $$\begin{aligned}&f(0,\eta ) = \eta \, {\text {erfc}}\left( \frac{\eta }{2}\right) + \frac{2}{\sqrt{\pi }}\left[ 1-\exp \left( -\frac{\eta ^2}{4}\right) \right] , \end{aligned}$$

8 $$\begin{aligned}&g(0,\eta ) = c\left( \eta \, {\text {erfc}}\left( \frac{\eta }{2}\right) + \frac{2}{\sqrt{\pi }}\left[ 1-\exp \left( -\frac{\eta ^2}{4}\right) \right] \right) , \end{aligned}$$

9 $$\begin{aligned}&\theta (0,\eta ) = {\text {erfc}}\left( \frac{\sqrt{Pr}\eta }{2}\right) , \end{aligned}$$

10 $$\begin{aligned}&\phi (0,\eta ) = {\text {erfc}}\left( \frac{\sqrt{Sc}\eta }{2}\right) . \end{aligned}$$

## Bivariate interpolated spectral relaxation method (BI-SRM)

In this section we introduce the Bivariate Interpolated Spectral Relaxation Method (BI-SRM) for solving the system of nonlinear partial differential equations (1)–(3). Applying the relaxation scheme Motsa et al. (2014a) to the system of nonlinear partial differential equations gives the following linear partial differential equations;

11 $$\begin{aligned}&f_{r+1}''' + \alpha _{1,r}f_{r+1}'' + \alpha _{2,r}f_{r+1}' + \alpha _{3,r}f_{r+1} - \xi (1-\xi ) \frac{\partial {f'_{r+1}}}{\partial {\xi }} = R_{1,r}, \end{aligned}$$

12 $$\begin{aligned}&g_{r+1}''' + \beta _{1,r}g_{r+1}'' + \beta _{2,r}g_{r+1}' + \beta _{3,r}g_{r+1} - \xi (1-\xi ) \frac{\partial {g'_{r+1}}}{\partial {\xi }} = R_{2,r}, \end{aligned}$$

13 $$\begin{aligned}&\theta _{r+1}'' + \sigma _{1,r}\theta _{r+1}' + \sigma _{2,r}\theta _{r+1} - {\text {Pr}}\xi (1-\xi ) \frac{\partial {\theta _{r+1}}}{\partial {\xi }} = R_{3,r}, \end{aligned}$$

14 $$\begin{aligned}&\phi _{r+1}'' + \omega _{1,r}\phi _{r+1}' + \omega _{2,r}\phi _{r+1} - {\text {Sc}}\xi (1-\xi )\frac{\partial {\phi _{r+1}}}{\partial {\xi }} = R_{4,r}, \end{aligned}$$

subject to

15 $$\begin{aligned}&f_{r+1}(\xi ,0) = g_{r+1}(\xi ,0) = 0, \;\;\; f'_{r+1}(\xi ,0) = \theta _{r+1}(\xi ,0) = \phi _{r+1}(\xi ,0) = 1,\nonumber \\&f'_{r+1}(\xi ,\infty ) = g'_{r+1}(\xi ,\infty ) =\theta _{r+1}(\xi ,\infty ) = \phi _{r+1}(\xi ,\infty ) = 0, \nonumber \\&g'_{r+1}(\xi ,0) = c \end{aligned}$$

where the variable coefficients are given by

$$\begin{aligned}&\alpha _{1,r}=\frac{1}{2}\eta (1-\xi ) + \xi g_r,\;\;\; \alpha _{2,r} = -\xi (M^2+\lambda ), \;\; \alpha _{3,r} = 0, \\&\beta _{1,r} = \frac{1}{2}\eta (1-\xi ) + \xi f_r, \;\;\; \beta _{2,r} = -\xi (M^2+\lambda ), \;\;\; \beta _{3,r} = 0, \\&\sigma _{1,r} = {\text {Pr}}\left( \frac{1}{2}\eta (1-\xi ) + \xi (f_r + g_r)\right) , \;\; \sigma _{2,r} = 0 \\&\omega _{1,r} = {\text {Sc}}\left( \frac{1}{2}\eta (1-\xi ) + \xi (f_r+g_r)\right) , \;\;\; \omega _{2,r} = -\gamma \, {\text {Sc}}\, \xi \\&R_{1,r} = \xi (f')_r^2 - \xi f_rf_r'', \;\; R_{2,r} = \xi (g')_r^2 -\xi g_rg_r'', \;\; R_{3,r} = 0, \;\; R_{4,r} = 0\\ \end{aligned}$$

and *r* and $r+1$ denote previous and current iterations respectively. The system of linear partial differential equations (11)–(14) is discretised using the Chebyshev spectral collocation both in space ($\eta$) and time ($\xi$) directions. The Chebyshev collocation method is valid in the domain $[-1,1]$ in space and time. Therefore, the physical region, $\xi \in [0,1]$ is converted to the region $t \in [-1,1]$ using a linear transformation and similarly, $\eta \in [0,L_{\infty }]$ is converted to the region $x \in [-1,1]$. The system of linear partial differential equations (11)–(14) is decoupled. Therefore, each equation can be solved independently of the other equations in the system. We assume that the solution to Eq. (11) can be approximated by a bivariate Lagrange interpolation polynomial of the form

16 $$\begin{aligned} f(\eta ,\xi )\approx \sum _{p=0}^{N_x}\sum _{j=0}^{N_t} f(x_p,t_j)L_p(x)L_j(t), \end{aligned}$$

which interpolates $f(\eta ,\xi )$ at selected points in both the $\eta$ and $\xi$ directions defined by

17 $$\begin{aligned} \{x_p\} = \left\{ \cos \left( \frac{\pi p}{N_x} \right) \right\} _{p=0}^{N_x},\;\;\;\;\; \{t_j\} = \left\{ \cos \left( \frac{\pi j}{N_t} \right) \right\} _{j=0}^{N_t}. \end{aligned}$$

The Chebyshev–Gauss–Lobatto grid points (17) ensures that there is a simple conversion of the continuous derivatives, in both space and time, to discrete derivatives at the grid points. The characteristic Lagrange cardinal polynomial $L_p(x)$ is defined as

18 $$\begin{aligned} L_p(x) = \mathop{\mathop{\prod}\limits_{p = 0}}\limits_{p \ne s}^{N_x} \frac{x - x_s}{x_p - x_s}, \end{aligned}$$

where

19 $$\begin{aligned} L_p(x_s) = \delta _{ps} = \left\{ \begin{array}{cc} 0\,\, &{}\,{\text{ if}}\,\, p \ne s \\ 1\,\, &{}\,{\text{ if }}\,\, p = s \end{array} \right. \end{aligned}$$

Similarly, we define the function $L_j(t)$. Equation (16) is then substituted into Eq. (11). An important step in the implementation of the solution procedure is the evaluation of the derivatives of $L_p(x)$ and $L_j(t)$ with respect to *x* and *t* respectively. The derivative of $f(\eta ,\xi )$ with respect to $\xi$ at the Chebyshev–Gauss–Lobatto points $(x_s,t_i)$, is computed as

20 $$\begin{aligned} \left. \frac{\partial f}{\partial \xi } \right| _{(x_s,t_i)}&=2\sum _{p = 0}^{N_x} \sum _{j=0}^{N_t} f(x_p,t_j) L_p(x_s)\frac{dL_j(t_i)}{dt} \end{aligned}$$

21 $$\begin{aligned}&= 2\sum _{j=0}^{N_t}d_{ij}f(x_s,t_j)= \sum _{j=0}^{N_t}d_{ij}{} {\mathbf{F}} _j \end{aligned}$$

where $d_{ij} = \frac{dL_j(t_i)}{dt}$ are the entries of the standard first derivative Chebyshev differentiation matrix ${\mathbf{d}} = [d_{ij}]$ of size $(N_t+1)\times (N_t+1)$ as defined in Trefethen (2000) for $i,j = 0,1,\ldots ,N_t$. Similarly, we compute the derivative of $f(\eta ,\xi )$ with respect to $\eta$ at the Chebyshev-Gauss-Lobatto points $(x_s,t_i)$, as follows

22 $$\begin{aligned} \left. \frac{\partial f}{\partial \eta } \right| _{(x_s,t_i)}&=\left( \frac{2}{L_{\infty }}\right) \sum _{p = 0}^{N_x}\sum _{j=0}^{N_t}f(x_p,t_j)\frac{dL_p(x_s)}{dx}L_j(t_i) \end{aligned}$$

23 $$\begin{aligned}&= \left( \frac{2}{L_{\infty }}\right) \sum _{p=0}^{N_x} D_{sp}f(x_p,t_i)= {\mathbf{D}}{\mathbf{F}} _{i}, \end{aligned}$$

where $D_{sp} = \frac{dL_p(x_s)}{dx}$, are the entries of the standard first derivative Chebyshev differentiation matrix of size $(N_x+1)\times (N_x+1)$. Therefore, an *n*th order derivative with respect to $\eta$ is given by

24 $$\begin{aligned} \left. \frac{\partial ^n f}{\partial \eta ^n} \right| _{(x_s,t_j)}=\left( \frac{2}{L_{\infty }}\right) ^n \sum _{p = 0}^{N_x}D^n_{sp}f(x_p,t_i)={\mathbf{D}}^n {\mathbf{F}} _{i},\;\;\;i = 0,1,2,\ldots ,N_x, \end{aligned}$$

The vector ${\mathbf{F}} _{i}$ is defined as

25 $$\begin{aligned} {\mathbf{F}} _{i} = [f_{i}(x_0), f_{i}(x_1), \ldots ,f_{i}(x_{N_x})]^T. \end{aligned}$$

where the superscript *T* denotes matrix transpose. Collocating using Eqs. (24) and (21) on (11), we get

26 $$\begin{aligned} \left[ {\mathbf{D}}^3 + \pmb {\alpha }_{1,r}{} {\mathbf{D}}^2 + \pmb {\alpha }_{2,r} {\mathbf{D}}+ \pmb {\alpha }_{3,r}\right] {\mathbf{F}} _{r+1,i} - \xi _i(1-\xi _i) \sum _{j=0}^{N_t} d_{ij}{} {\mathbf{D}}{} {\mathbf{F}} _{r+1,j} = {\mathbf{R}} _{1,r},\;\;\;i = 0,1,2,\ldots ,N_t, \end{aligned}$$

where $\pmb {\alpha }_{v,r}$ ($v = 1,2,3$) is the diagonal matrix of the vector $[\alpha _{v,r}(x_0), \alpha _{v,r}(x_1), \ldots ,\alpha _{v,r}(x_{N_x}) ]^T$ and ${\mathbf{R}} _{1,r} = [R_{1,r}(x_0),R_{1,r}(x_1),\ldots ,R_{1,r}(x_{N_x}) ]^T$. The boundary equations are given by

27 $$\begin{aligned} f_{r+1,i}(x_{N_x}) = 0,\;\;\;f'_{r+1,i}(x_{N_x}) = 1,\;\;\;f'_{r+1,i}(x_{0}) = 0,\;\;\; \end{aligned}$$

The initial unsteady solution given by equation (7) corresponds to $$ t = t_{N_t} = -1$$. Therefore, we evaluate Eq. (26) for $$i = 0,1,\ldots ,N_t - 1$$. Equation (26) can be expressed as

28 $$\begin{aligned} \left[ {\mathbf{D}}^3 + \pmb {\alpha }_{1,r}{} {\mathbf{D}}^2 + \pmb {\alpha }_{2,r} {\mathbf{D}}+ \pmb {\alpha }_{3,r} \right] {\mathbf{F}} _{r+1,i} - \xi _i(1-\xi _i) \sum _{j=0}^{N_t-1} d_{ij}{} {\mathbf{D}}{} {\mathbf{F}} _{r+1,j} = {\mathbf{R}} _{1,i},\;\;\;i = 0,1,2,\ldots ,N_t, \end{aligned}$$

where

$$\begin{aligned} {\mathbf{R}} _{1,i} = {\mathbf{R}} _{1,r} + \xi _i(1-\xi _i)d_{iN_t}{} {\mathbf{D}}{} {\mathbf{F}} _{N_t}, \end{aligned}$$

and ${\mathbf{F}} _{N_t}$ is the known initial unsteady solution given by equation (7). Imposing boundary conditions for $i = 0,1,\ldots ,N_t - 1$, Eq. (28) can be expressed as the following $N_t(N_x+1) \times N_t(N_x+1)$ matrix system

29 $$\begin{aligned} \left[\begin{array}{cccc} A_{0,0}&A_{0,1}&\cdots&A_{0,N_t-1} \\ A_{1,0}&A_{1,1}&\cdots&A_{1,N_t-1} \\ \vdots&\vdots&\ddots&\vdots \\ A_{N_t-1,0}&A_{N_t-1,1}&\cdots&A_{N_t-1,N_t-1} \end{array}\right] \left[\begin{array}{c} {\mathbf{F}} _{0}\\ {\mathbf{F}} _{1}\\ \vdots \\ {\mathbf{F}} _{N_t-1} \end{array}\right] = \left[\begin{array}{c} {\mathbf{R}} _{1,0}\\ {\mathbf{R}} _{1,1}\\ \vdots \\ {\mathbf{R}} _{1,N_t-1}\\ \end{array}\right], \end{aligned}$$

where

30 $$\begin{aligned} A_{i,i}= {\mathbf{D}}^3 + \pmb {\alpha }_{1,r}{} {\mathbf{D}}^2 + \pmb {\alpha }_{2,r}{} {\mathbf{D}}+ \pmb {\alpha }_{3,r} - \xi _i(1-\xi _i)d_{ii}{} {\mathbf{D}} \end{aligned}$$

31 $$\begin{aligned} A_{i,j}&= - \xi _i(1-\xi _i)d_{ij}{} {\mathbf{D}},\;\;\; {\text{ when }} i \ne j, \end{aligned}$$

Imposing initial boundary conditions and applying the Chebyshev bivariate collocation as described above on Eqs. (12), (13) and (14) we get

32 $$\begin{aligned}&\left[ {\mathbf{D}}^3 + \pmb {\beta }_{1,r}{} {\mathbf{D}}^2 + \pmb {\beta }_{2,r}{} {\mathbf{D}}+ \pmb {\beta }_{3,r}\right] {\mathbf{G}} _{r+1,i} - \xi _i(1-\xi _i)\sum _{j=0}^{N_t-1}d_{ij}{} {\mathbf{D}}{} {\mathbf{G}} _{r+1,j} = {\mathbf{R}} _{2,i}, \end{aligned}$$

33 $$\begin{aligned}&\left[ {\mathbf{D}}^2 + \pmb {\sigma }_{1,r}{} {\mathbf{D}}+ \pmb {\sigma }_{2,r}\right] \pmb {\Theta }_{r+1,i} - {\text {Pr}}\xi _i(1-\xi _i)\sum _{j=0}^{N_t-1}d_{ij}\pmb {\Theta }_{r+1,j} = {\mathbf{R}} _{3,i}, \end{aligned}$$

34 $$\begin{aligned}&\left[ {\mathbf{D}}^2 + \pmb {\omega }_{1,r}{} {\mathbf{D}}+ \pmb {\omega }_{2,r}\right] \pmb {\Phi }_{r+1,i} - {\text {Sc}}\xi _i(1-\xi _i)\sum _{j=0}^{N_t-1}d_{ij}\pmb {\Phi }_{r+1,j} = {\mathbf{R}} _{4,i}, \end{aligned}$$

where

35 $$\begin{aligned}&{\mathbf{R}} _{2,i} = {\mathbf{R}} _{2,r} + \xi _i(1-\xi _i)d_{iN_t}{} {\mathbf{D}}{} {\mathbf{G}} _{N_t}, \end{aligned}$$

36 $$\begin{aligned}&{\mathbf{R}} _{3,i} = {\mathbf{R}} _{3,r} + {\text {Pr}}\xi _i(1-\xi _i)d_{iN_t}\pmb {\Theta }_{N_t}, \end{aligned}$$

37 $$\begin{aligned} {\mathbf{R}} _{4,i} = {\mathbf{R}} _{4,r} + {\text {Sc}}\xi _i(1-\xi _i)d_{iN_t}\pmb {\Phi }_{N_t}, \end{aligned}$$

and the vectors ${\mathbf{G}} _{N_t}$, $\pmb {\Theta }_{N_t}$ and $\pmb {\Phi }_{N_t}$ are the known initial unsteady solutions given by Eqs. (8), (9) and (10) respectively. Imposing boundary conditions for $i = 0,1,\ldots ,N_t - 1$, equations (32), (33) and (34) can be expressed as the following $N_t(N_x+1) \times N_t(N_x+1)$ matrix system

38 $$\begin{aligned} \left[ \begin{array}{cccc} B_{0,0} &{} B_{0,1} &{} \cdots &{} B_{0,N_t-1} \\ B_{1,0} &{} B_{1,1} &{} \cdots &{} B_{1,N_t-1} \\ \vdots &{} \vdots &{} \ddots &{} \vdots \\ B_{N_t-1,0} &{} B_{N_t-1,1} &{} \cdots &{} B_{N_t-1,N_t-1} \end{array}\right] \left[ \begin{array}{c} {\mathbf{G}} _{0}\\ {\mathbf{G}} _{1}\\ \vdots \\ {\mathbf{G}} _{N_t-1} \end{array}\right]= & {} \left[ \begin{array}{c} {\mathbf{R}} _{2,0}\\ {\mathbf{R}} _{2,1}\\ \vdots \\ {\mathbf{R}} _{2,N_t-1}\\ \end{array}\right] , \end{aligned}$$

39 $$\begin{aligned} \left[ \begin{array}{cccc} C_{0,0} &{} C_{0,1} &{} \cdots &{} C_{0,N_t-1} \\ C_{1,0} &{} C_{1,1} &{} \cdots &{} C_{1,N_t-1} \\ \vdots &{} \vdots &{} \ddots &{} \vdots \\ C_{N_t-1,0} &{} C_{N_t-1,1} &{} \cdots &{} C_{N_t-1,N_t-1} \end{array}\right] \left[ \begin{array}{c} \pmb {\Theta }_{0}\\ \pmb {\Theta }_{1}\\ \vdots \\ \pmb {\Theta }_{N_t-1} \end{array}\right]= & {} \left[ \begin{array}{c} {\mathbf{R}} _{3,0}\\ {\mathbf{R}} _{3,1}\\ \vdots \\ {\mathbf{R}} _{3,N_t-1}\\ \end{array}\right] , \end{aligned}$$

40 $$\begin{aligned} \left[ \begin{array}{cccc} E_{0,0} &{} E_{0,1} &{} \cdots &{} E_{0,N_t-1} \\ E_{1,0} &{} E_{1,1} &{} \cdots &{} E_{1,N_t-1} \\ \vdots &{} \vdots &{} \ddots &{} \vdots \\ E_{N_t-1,0} &{} E_{N_t-1,1} &{} \cdots &{} E_{N_t-1,N_t-1} \end{array}\right] \left[ \begin{array}{c} \pmb {\Phi }_{0}\\ \pmb {\Phi }_{1}\\ \vdots \\ \pmb {\Phi }_{N_t-1} \end{array}\right]= & {} \left[ \begin{array}{c} {\mathbf{R}} _{4,0}\\ {\mathbf{R}} _{4,1}\\ \vdots \\ {\mathbf{R}} _{4,N_t-1}\\ \end{array}\right] , \end{aligned}$$

where

41 $$\begin{aligned} B_{i,i}&= {\mathbf{D}}^3 + \pmb {\beta }_{1,r}{} {\mathbf{D}}^2 + \pmb {\beta }_{2,r}{} {\mathbf{D}}+ \pmb {\beta }_{3,r} - \xi _i(1-\xi _i)d_{ii}{} {\mathbf{D}}\end{aligned}$$

42 $$\begin{aligned} B_{i,j}&= - \xi _i(1-\xi _i)d_{ij}{} {\mathbf{D}},\;\;\; {\text{ when }} i \ne j, \end{aligned}$$

43 $$\begin{aligned} C_{i,i}&= {\mathbf{D}}^2 + \pmb {\sigma }_{1,r}{} {\mathbf{D}}+ \pmb {\sigma }_{2,r} - {\text {Pr}}\xi _i(1-\xi _i)d_{ii}{} {\mathbf{I}} \end{aligned}$$

44 $$\begin{aligned} C_{i,j}&= - {\text {Pr}}\xi _i(1-\xi _i)d_{ij}{} {\mathbf{I}} ,\;\;\; {\text{ when }} i \ne j, \end{aligned}$$

45 $$\begin{aligned} E_{i,i}&= {\mathbf{D}}^2 + \pmb {\omega }_{1,r}{} {\mathbf{D}}+ \pmb {\omega }_{2,r} - {\text {Sc}}\xi _i(1-\xi _i)d_{ii}{} {\mathbf{I}} \end{aligned}$$

46 $$\begin{aligned} E_{i,j}&= - {\text {Sc}}\xi _i(1-\xi _i)d_{ij}{} {\mathbf{I}} ,\;\;\; {\text{ when }} i \ne j, \end{aligned}$$

and ${\mathbf{I}}$ is the standard $(N_x+1) \times (N_x+1)$ identity matrix. We obtain the numerical solutions for $g(\eta ,\xi )$, $\theta (\eta ,\xi )$ and $\phi (\eta ,\xi )$ by solving matrix equations (38), (39) and (40) iteratively for $r = 1,2,\ldots M$, where *M* is the number of iterations to be used. Equations (8), (9) and (10) are used as initial guesses.

## Results and discussion

In this section we present the numerical solutions of the three dimensional unsteady three dimensional magneto-hydrodynamic flow and mass transfer in a porous media obtained using the BI-SQLM algorithm. In our computations the $\eta$ domain was truncated to $\eta _{\infty }=20$. This value gave accurate results for all the quantities of physical interest. To get accurate solutions, $N_x = 60$ collocation points were used to discretize the space variable $\eta$ and only $N_t = 10$ collocation points were enough for the time variable $\xi$.

As earlier mentioned, this problem has been solved before by Motsa et al. (2014a) using the spectral relaxation method (SRM), spectral quasilinearization method (SQLM) and the Keller-box method. The results from their paper combined with the present results of the BI-SRM are shown in Table 1. It can be observed from the table that the Keller-box method takes a significant amount of computational time than the SRM and SQLM. This is because the Keller-box is entirely based on finite difference schemes while the SRM and SQLM only uses finite differences in the time variable. In the space variable both the SRM and SQLM use spectral method. It is well documented from literature that spectral methods converge very fast when the solution is smooth. This brought about the idea of using spectral methods in both space and time to increase efficiency. The BI-SRM discretizes both the space and time domains using spectral methods. From the results shown in the table it is evident that the BI-SRM is by far superior than the other methods in terms of computational time taken to reach the same level of accuracy. In Table 1, we also show the number of grid points required by each of the methods to discretize in time. All the finite difference based discretizations required 2000 grid points compared to the spectral discretization of the BI-SRM which required only 10 grid points to reach the same level of accuracy.

**Table 1:** Values of $f''(0,\xi )$, $g''(0,\xi )$, $\theta '(0,\xi )$ and $\phi '(0,\xi )$ when $\lambda = 0.5, M = 2, c = 0.5, Sc = \gamma = 1, Pr = 1.5$

$\xi$	BI-SRM	SRM	SQLM	Keller-box
	($N_t = 10$)	($N_t = 2000$)	($N_t = 2000$)	($N_t = 2000$)
	$$ f''(0,\xi )$$			
0.1	−0.851257	−0.851257	−0.851257	−0.851257
0.3	−1.316705	−1.316705	−1.316705	−1.316705
0.5	−1.685306	−1.685306	−1.685306	−1.685306
0.7	−1.992608	−1.992608	−1.992608	−1.992608
0.9	−2.259335	−2.259335	−2.259335	−2.259335
	$$ g''(0,\xi )$$			
0.1	−0.417150	−0.417150	−0.417150	−0.417150
0.3	−0.639602	−0.639602	−0.639602	−0.639602
0.5	−0.817649	−0.817649	−0.817649	−0.817649
0.7	−0.966603	−0.966603	−0.966603	−0.966603
0.9	−1.095983	−1.095983	−1.095983	−1.095983
	$$ \theta '(0,\xi )$$			
0.1	−0.710882	−0.710882	−0.710882	−0.710882
0.3	−0.742842	−0.742842	−0.742842	−0.742842
0.5	−0.765244	−0.765244	−0.765244	−0.765244
0.7	−0.777270	−0.777270	−0.777270	−0.777270
0.9	−0.770807	−0.770807	−0.770807	−0.770807
	$$ \phi '(0,\xi )$$			
0.1	−0.634443	−0.634443	−0.634443	−0.634443
0.3	−0.766867	−0.766867	−0.766867	−0.766867
0.5	−0.891207	−0.891207	−0.891207	−0.891207
0.7	−1.010045	−1.010045	−1.010045	−1.010045
0.9	−1.125549	−1.125549	−1.125549	−1.125549
CPU time	0.47	18.90	83.24	900.30

The grid independence test for the algorithm is shown in Table 2. The skin friction values, Nusselt number and Sherwood numbers are the variables used in carrying out the grid independence test in Table 2.

**Table 2:** Values of $f''(0,\xi )$, $g''(0,\xi )$, $\theta '(0,\xi )$ and $\phi '(0,\xi )$ when $\lambda = 0.5, M = 2, c = 0.5, Sc = \gamma = 1, Pr = 1.5$

$\xi \backslash N_t$	5	10	15	20
	$$ f''(0,\xi )$$			
0.1	−0.85118289	−0.85125725	−0.85125723	−0.85125724
0.3	−1.31678885	−1.31670509	−1.31670508	−1.31670508
0.5	−1.68525477	−1.68530619	−1.68530619	−1.68530619
0.7	−1.99262557	−1.99260827	−1.99260827	−1.99260827
0.9	−2.25932899	−2.25933501	−2.25933501	−2.25933501
	$$ g''(0,\xi )$$			
0.1	−0.41712280	−0.41715041	−0.41715040	−0.41715040
0.3	−0.63962554	−0.63960199	−0.63960200	−0.63960200
0.5	−0.81764133	−0.81764898	−0.81764898	−0.81764898
0.7	−0.96660357	−0.96660340	−0.96660340	−0.96660340
0.9	−1.09598187	−1.09598304	−1.09598304	−1.09598304
	$$ \theta '(0,\xi )$$			
0.1	−0.71037577	−0.71087263	−0.71088151	−0.71088162
0.3	−0.74357007	−0.74283215	−0.74284211	−0.74284190
0.5	−0.76493472	−0.76524664	−0.76524370	−0.76524351
0.7	−0.77722565	−0.77727704	−0.77727014	−0.77727005
0.9	−0.77086219	−0.77080729	−0.77080662	−0.77080662
	$$ \phi '(0,\xi )$$			
0.1	−0.63444437	−0.63444336	−0.63444326	−0.63444326
0.3	−0.76685596	−0.76686699	−0.76686689	−0.76686689
0.5	−0.89121805	−0.89120664	−0.89120666	−0.89120666
0.7	−1.01004174	−1.01004487	−1.01004494	−1.01004494
0.9	−1.12555031	−1.12554912	−1.12554913	−1.12554913
CPU time	0.13	0.47	1.25	2.47

**Fig. 1:**
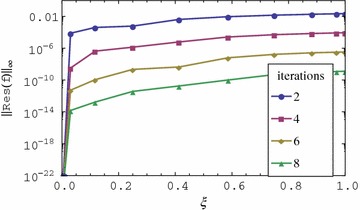
Residual graph of $f(\eta ,\xi )$

**Fig. 2:**
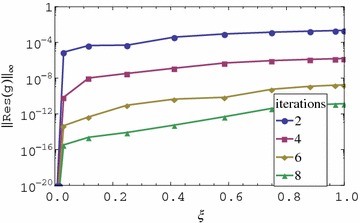
Residual graph of $g(\eta ,\xi )$

**Fig. 3:**
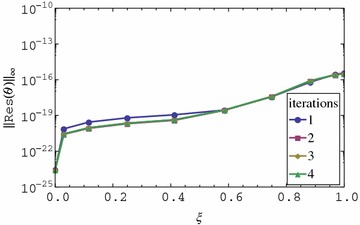
Residual graph of $\theta (\eta ,\xi )$

**Fig. 4:**
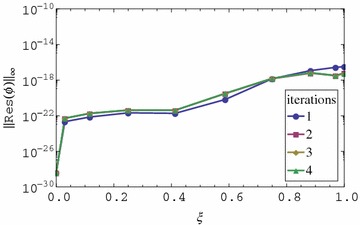
Residual graph of $\phi (\eta ,\xi )$

The residual error graphs of Eqs. (1)–(3) are presented in Figs. 1, 2, 3 and 4 respectively. In Figs. 1 and 2, we observe that the residual error is reduced with an increase in the iterations of the scheme. The rate of reduction of the residual error appears to be linear. The residual error is minimum at $\xi = 0$ and is increased sharply near 0 until a certain level is reached after which it is almost constant. The residual error appears to be nearly uniform in $0 < \xi \le 1$ or increases only slightly. It is also observed that the order of magnitude of the residual error can be seen to be small in the $0 \le \xi \le 1$ interval. Lastly, after only two iterations the residual error appears to be less than 0.01 in the entire range of $\xi$. The small residual error using only a few iteration points to the accuracy of the method. This error can be decreased at a linear rate with an increase in the number of iterations. The decrease in the error with additional iterations suggests that the iteration scheme converges. It should be noted that when $\xi = 0$, governing equations reduce to a linear system that can be solved directly using the spectral collocation method with discretization only in $\eta$ without the use of relaxation and iterations. This explains why the best accuracy is observed at $\xi = 0$. The near uniformity of the residual error in $0 < \xi \le 1$ can be attributed to the use of Lagrange polynomial basis functions whose error is known to be uniformly distributed in the interpolating region. We can therefore conclude that the method gives accurate results, the rate of convergence of the method is linear and that the method requires only a few iterations to give very accurate results.

We observe that the residual error for the first iteration appears to be very small in Figs. 3 and 4. The residual error is the same for all iterations greater than one. The residual error increases with an increase in $\xi$. We also observe that the residual error is smaller than the one for *f* and *g* even when fewer iterations are used. The observation that the residual error for the momentum and energy is small even for the 1st iteration is perhaps the most interesting finding of the study. This means that when using the proposed approach, the best possible results that can be achieved by the method can be obtained after using just one iteration. Further increase in the number of iterations doesn’t improve the accuracy of the solution. After one iteration of the momentum equations for *f* and *g* the energy and mass transfer equations reduce to linear homogeneous equations whose solution appears to be marginally influenced by variations in $f_r$ and $g_r$ for $r > 1$. Since with just one iteration we obtain extremely accurate results for $\theta$ and $\phi$, the implication is that in solving for energy and momentum equations for such a problem, it is not necessary to iterate. It is enough to just use the initial approximation. Is is worth noting that the energy and mass transfer equations are homogeneous equations in $\theta$ and $\phi$ respectively. It is possible that the findings obtained in this study are only applicable in such equations. This has to be investigated further.

**Fig. 5:**
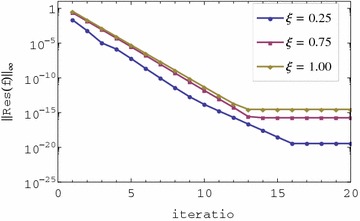
Convergence graph of $f(\eta ,\xi )$

**Fig. 6:**
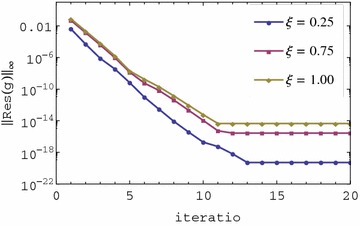
Convergence graph of $g(\eta ,\xi )$

The convergence graphs of Eqs. (1)–(3) are presented in Figs. 5, 6 respectively. In Figs. 5 and 6, the residual error decreases linearly with an increase in the number of iterations. The residual error is smallest when $\xi$ is near zero and largest when $\xi$ is large. This is seen from the convergence level which is near $10^{-20}$ for $\xi = 0.25$ and about $10^{-15}$ for $\xi = 0.75$. The slope of the residual error graphs is the same for all values of $\xi$. Full convergence is achieved after 13 iterations for both $\xi = 0.75$ and $\xi = 1$. For $\xi = 0.25$ full convergence is achieved after 16 iterations but at a much smaller magnitude of residual error. The decrease in the residual error with increase in iterations suggests that the iteration scheme converges. Small residual error near zero suggests that best accuracy (after full convergence) is observed near zero. The method converges (fewer iterations needed to attain full convergence) at or near $\xi = 1$. However, the convergence efficiency doesn’t translate to better accuracy because, as can be seen for the case of $\xi$ values near zero, the convergence level is $10^{-16}$. The same slope for all the graphs means that the convergence rates of the method is the same for all values of $\xi$. The method is convergent and very accurate in whole time domain $\xi \in [0,1]$ which translates to $\tau \in [0,\infty )$. The method converges with nearly the same convergence rate for all values of $\xi$. The method gives the best accuracy near $\xi = 0$ and less accurate, comparatively, at or near $\xi = 1$. We note that even at $\xi = 1$, the method gives very accurate results with a residual error norm of about $10^{-15}$. This is one of the highlights of this investigation.

The accuracy of the solutions for energy and mass transfer equations are not dependent on successive relaxation or iterations of the momentum equations since the convergence of the solutions doesn’t improve at all with an increase in the number of iterations. Hence, the results for $\theta (\eta )$ are not dependent on successive approximations for $f(\eta )$ and $g(\eta )$. It was observed that it is enough to use one iteration of $f(\eta )$ to give accurate result for $\theta (\eta )$.

In Table 1, we present the results we obtained using the algorithm. The skin friction values, Nusselt number and Sherwood number for various values of $\xi$ are displayed in Table 1. The results obtained using the method match those obtained using other methods. Computing time for the BI-SRM is much smaller than the other methods it is compared with. The method gives valid results when used with few collocation points. In particular, the BI-SRM requires only ten grid points to achieve the same valid results. The table validates the results obtained in this study. BI-SRM is computationally fast in generating valid results when compared with the SRM, SQLM and Keller-Box. We can infer that the BI-SRM is better than finite differences coupled SRM in terms of computational speed and accuracy and the better accuracy could be the result of applying spectral collocation with uniform accuracy level in both $\eta$ and $\xi$ directions.

In Tables 3 and 4, the residual errors and convergence rates of *g* and *g* when $\lambda = 0.5, M = 2, c = 0.5, Sc = \gamma = 1, Pr = 1$ are displayed. We observe that the convergence rate is linear and the residual error is smaller near $\xi = 0$.

**Table 3:** The residual errors and convergence rates of *f* when $\lambda = 0.5, M = 2, c = 0.5, Sc = \gamma = 1, Pr = 1.5$

Iter.	$\Vert Res({\mathbf{f}} )\Vert _{\infty }$			Convergence rates		
	$\xi = 0.25$	$\xi = 0.75$	$\xi = 1.00$	$\xi = 0.25$	$\xi = 0.75$	$\xi = 1.00$
1	$2.14\times 10^{-2}$	$2.36\times 10^{-1}$	$3.72\times 10^{-1}$	1.14	1.00	0.98
2	$6.03\times 10^{-4}$	$1.43\times 10^{-2}$	$2.24\times 10^{-2}$	0.50	1.01	1.01
3	$1.05\times 10^{-5}$	$8.58\times 10^{-4}$	$1.43\times 10^{-3}$	1.53	1.00	1.00
4	$1.36\times 10^{-6}$	$5.04\times 10^{-5}$	$8.85\times 10^{-5}$	1.06	1.01	1.00
5	$5.95\times 10^{-8}$	$2.98\times 10^{-6}$	$5.52\times 10^{-6}$	0.97	1.02	1.00
6	$2.18\times 10^{-9}$	$1.70\times 10^{-7}$	$3.40\times 10^{-7}$	0.99	1.00	1.00
7	$8.84\times 10^{-11}$	$9.06\times 10^{-9}$	$2.07\times 10^{-8}$	0.95	1.00	1.00
8	$3.75\times 10^{-12}$	$4.85\times 10^{-10}$	$1.27\times 10^{-9}$	0.85	0.99	1.00

**Table 4:** The residual errors and convergence rates of *g* when $\lambda = 0.5, M = 2, c = 0.5, Sc = \gamma = 1, Pr = 1.5$

Iter.	$\Vert Res({\mathbf{g}} )\Vert _{\infty }$			Convergence Rates		
	$\xi = 0.25$	$\xi = 0.75$	$\xi = 1.00$	$\xi = 0.25$	$\xi = 0.75$	$\xi = 1.00$
1	$4.17\times 10^{-3}$	$4.93\times 10^{-2}$	$7.83\times 10^{-2}$	0.94	1.01	0.99
2	$4.96\times 10^{-5}$	$1.40\times 10^{-3}$	$2.21\times 10^{-3}$	0.75	1.03	1.04
3	$7.66\times 10^{-7}$	$3.81\times 10^{-5}$	$6.39\times 10^{-5}$	1.26	1.13	1.18
4	$3.38\times 10^{-8}$	$9.21\times 10^{-7}$	$1.58\times 10^{-6}$	1.07	0.76	0.56
5	$6.56\times 10^{-10}$	$1.38\times 10^{-8}$	$2.01\times 10^{-8}$	0.83	0.68	0.92
6	$9.49\times 10^{-12}$	$5.75\times 10^{-10}$	$1.78\times 10^{-9}$	1.00	1.27	1.18
7	$2.82\times 10^{-13}$	$6.58\times 10^{-11}$	$1.93\times 10^{-10}$	0.89	1.08	1.04
8	$8.39\times 10^{-15}$	$4.19\times 10^{-12}$	$1.39\times 10^{-11}$	0.95	1.00	1.00

## Conclusion

The aim of this work was to describe the bivariate spectral relaxation method for systems of coupled partial differential equations. This technique extends the previous spectral relaxation method of Motsa et al. (2014a) to allow for discretization of both time and space derivatives using spectral collocation methods. The following conclusions can be drawn regarding this method;

- The method gives accurate results in the whole space and time domains $\xi \in [0,1]$ and $\tau \in [0,\infty )$ with residual errors rapidly approaching zero.
- The application of spectral collocation to both time and space derivatives ensures that the method performs significantly better than the SRM and the Keller-Box method in terms of computational time.
- The algorithm involves the usage of known formulas for discretization using Chebyshev spectral collocation.

In future we intend to show that the technique can be extended to coupled non-linear systems in three-dimensions.
